# A reproducible and repeatable digital method for quantifying nasal and sinus airway changes following suture palatine expansion

**DOI:** 10.1007/s11325-022-02691-2

**Published:** 2022-08-17

**Authors:** Georgia Tzironi, Álvaro Zubizarreta-Macho, Clara Guinot-Barona, Jorge Alonso Pérez-Barquero, Santiago Arias, Purificación Vicente-Galindo, Alberto Albaladejo Martínez

**Affiliations:** 1 Department of Surgery, Faculty of Medicine, University of Salamanca, 37008 Salamanca, Spain; 2 Department of Endodontics, Faculty of Health Sciences, Alfonso X El Sabio University, Avda. Universidad, 1, Villanueva de la Cañada, 28691 Madrid, Spain; 3 Department of Orthodontics, Faculty of Medicine and Health Sciences, Catholic University of Valencia, 46001 Valencia, Spain; 4 Department of Stomatology, Faculty of Medicine and Dentistry, University of Valencia, 46010 Valencia, Spain; 5 Department of Dentistry, Faculty of Health Sciences, CEU Cardenal Herrera University, CEU Universities, Moncada, Spain

**Keywords:** Orthodontics, Suture palatal expansion, Maxillary sinus, Nasal airway, Rapid maxillary expansion

## Abstract

**Purpose:**

The airway complex is modified by palatine expansion. Computer tomography has been used in the past to determine the change in volume, but there was a lack of a specific, reproducible method for this purpose. The present study sought to determine the accuracy, reproducibility, and repeatability of an innovative digital measurement technique for analyzing the volume of maxillary and nasal sinus airways following suture palatine expansion performed with the Hyrax disyuntor appliance.

**Methods:**

Patients underwent preoperative and postoperative cone-beam computed tomography (CBCT) scans. The datasets were subsequently uploaded into a digital treatment planning software to record the volume of the right and left maxillary sinus, as well as the nasal and maxillary sinus airway complex. The Gage Repeatability & Reproducibility statistical analysis methodology was used to evaluate the repeatability and reproducibility of this measurement technique when measuring the volume of maxillary and nasal sinus airways following suture palatine expansion with the Hyrax disyuntor appliance. Additionally, comparative analysis between preoperative and postoperative measures was performed using Student’s *t*-test for statistical analysis.

**Results:**

In 5 patients, paired *t*-tests found statistically significant differences before and after treatment in the volumes of the left maxillary sinus (*p* = 0.002), right maxillary sinus (*p* = 0.001), and nasal and maxillary sinus airway complex (*p* = 0.005) after suture palatine expansion with the Hyrax disyuntor appliance.

**Conclusion:**

The proposed digital technique is an accurate, repeatable, and reproducible measurement technique for analyzing the volume of maxillary and nasal sinus airways following suture palatine expansion using the Hyrax disyuntor.

## Introduction

Maxillary transverse deficiency is a type of dental malocclusion that affects 9.4% of the entire population and almost 30% of adult patients [1]. It can lead to serious respiratory problems as a result of the nasal constriction that tends to accompany it [2]. In patients who are still developing, the condition is easily treated using conventional rapid palatal expansion techniques [3]. Rapid palatal expansion opens the mid-palatal suture, increasing the width of the maxilla in order to correct the transverse deficiency. In 2012, Torre and Alarcón concluded that rapid maxillary expansion (RME) using the Hyrax appliance in 10-year-old children with oral breathing was an efficient approach that improved nasal airflow, with results remaining stable after 6-month and one-year follow-up [4]. Per the results of a systematic review conducted by Baratieri et al., changes in nasal breathing after RME remain stable for at least 11 months after orthopedic treatment [5]. The volume of the maxillary and nasal sinus airways has previously been assessed using lineal measurement procedures [6], lateral and antero-posterior radiographs [6], mathematical equations [7], computer tomography (CT) [8], and acoustic rhinometry [9]; however, many of these techniques do not enable accurate measurement of the total volume of the maxillary and nasal sinus airways, or they entail a steep learning curve. Therefore, digital measurement techniques have been suggested for use in measuring the volume of anatomical structures [10].

The present study sought to measure the accuracy, repeatability, and reproducibility of an innovative digital measurement technique for analyzing the volume of maxillary and nasal sinus airways after suture palatine expansion using the Hyrax disyuntor appliance, with a null hypothesis (*H*_0_) stating that the new digital measurement technique would not record accurate, repeatable, and reproducible volumes of maxillary and nasal sinus airways following suture palatine expansion with the Hyrax disyuntor appliance.

## Methods and materials

### Study design

This in vitro study was performed at the Department of, from November 2020 to March 2021 with the authorization of the Ethical Committee of the. All patients provided their informed consent for the preoperative and postoperative CBCT scans.

### Experimental procedure

**Fig. 1:**
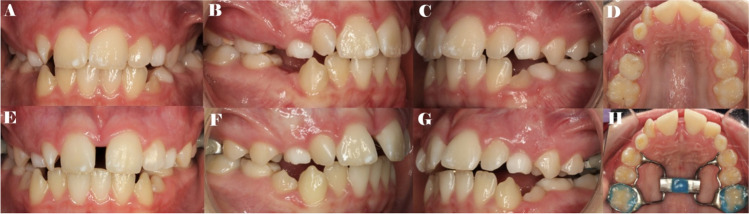
**A** Preoperative intraoral images of the frontal, **B** right lateral, **C** left lateral, and **D** occlusal view. **E** Postoperative intraoral images of frontal, **F** right lateral, **G** left lateral, and **G** occlusal view with the Hyrax disyuntor appliance

Patients aged from 10 to 13 years old with Class II molar and canine relationship, presenting with maxillary compression and without any nasal or nasopharyngeal airway obstruction, were consecutively selected and submitted to suture palatine expansion using the Hyrax disyuntor appliance over a mean activation time of 3 weeks and mean stabilization time of 9 months, using two turns per day, once in the morning and once at night at 0.5 mm every day (Fig. 1). The Hyrax disyuntor appliance was first indicated for functional reasons to correct Class II malocclusions, although it can also increase the nasal cavity and the maxillary sinus airway volume, lowering the risk of sleep disorders. Additionally, D’Souza et al. reported that transverse maxillary deficiency can lead to aesthetic and functional impairment, potentially leading to several clinical issues including functional or positional mandibular deviations, asymmetrical facial growth, or altered dentofacial aesthetics [11]; therefore, rapid palatal expansion appliances also influence functionality and aesthetics, as well as preventing further pathologies. All of the patients’ parents provided their informed consent for orthodontic treatment. The inclusion criteria were growing patients with late mixed dentition who had never undergone previous orthodontic treatment and had no history of systemic conditions that could interfere with bone metabolism.

### Measurement procedure

**Fig. 2:**
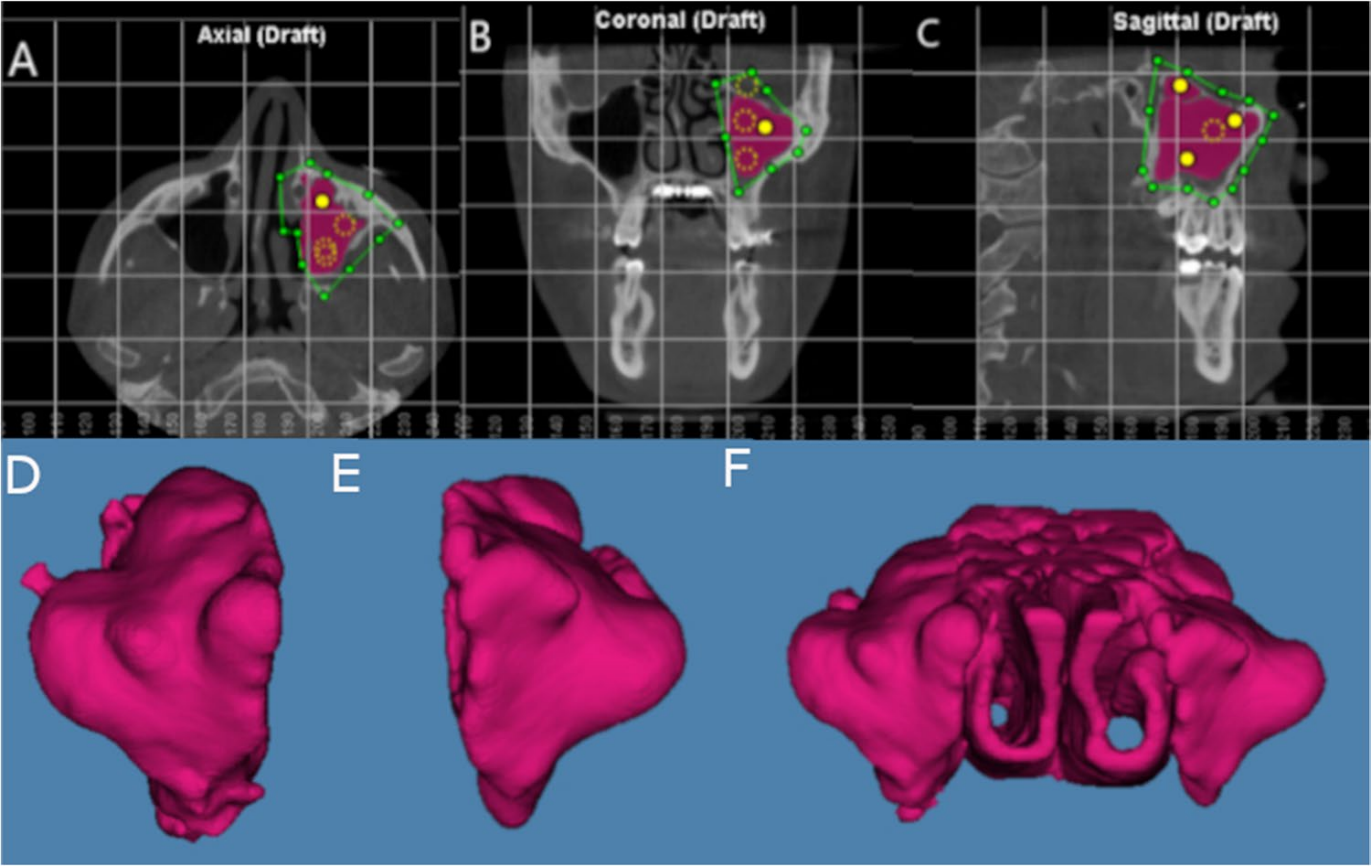
**A** Axial, **B** coronal, and **C** sagittal plane of the CBCT scans. The green line indicates the selected area, yellow points represent the air density, and the purple area shows the volume airway of the left maxillary sinus. **D** Volumetric assessment of the right maxillary sinus, **E** left maxillary sinus, and **F** nasal and maxillary sinus airway complex

All patients underwent cone-beam computed tomography (CBCT) scans (WhiteFox, Satelec, Merignac, France) prior to and following suture palatine expansion with the Hyrax disyuntor appliance, which was used with the following parameters: 105.0-kV peak, 7.20 s, 8.0 mA, and a field of view of 15 mm × 13 mm, by lining the Frankfort plane up with the floor using frontal and chin support. The CBCT scans (WhiteFox, Satelec, Merignac, France) taken preoperatively and postoperatively were then imported into digital software for therapeutic planning (Dolphin Imaging, Dolphin Imaging & Management Solutions, Chatsworth, CA, USA) to accurately measure the volume of the left maxillary sinus, right maxillary sinus, and the nasal and maxillary sinus airway complex. Prior to measuring the airway volumes, clinicians identified the anatomical area in the axial (Fig. 2A), coronal (Fig. 2B), and sagittal (Fig. 2C) planes and ensured accurate measurement of air density by placing reference points within the selected area. The most extreme point in each plane was identified, and a drawing was made using these points so as to include the area chosen for measurement. Then, the appropriate sensitivity was selected within the program so that all the airway points within the selected field were included. Finally, each drawing was measured digitally. It is important to mention that the same sensitivity was used for all airway measurements in each scan.

Subsequently, the software program (Dolphin Imaging, Dolphin Imaging & Management Solutions, Chatsworth, CA, USA) Airway Measurement tool was used to measure the volumes of the right maxillary sinus (Fig. 2D), left maxillary sinus (Fig. 2E), and nasal and maxillary sinus airway complex (Fig. 2F) following the palatine expansion procedure.

### Validation of technique repeatability and reproducibility

In order to validate the repeatability and reproducibility of this digital measurement technique to quantify the volumes of both maxillary sinuses and the maxillary sinus airway complex, two operators (Operator A and Operator B) randomly (Epidat 4.1, Galicia, Spain) selected and measured these variables three times after suture palatine expansion using the Hyrax disyuntor appliance, before carrying out a Gage R&R statistical analysis.

### Statistical tests

Descriptive statistics were expressed in mean values and standard deviations (SD). The Shapiro–Wilk normality test was also carried out. Comparative analysis of the right maxillary sinus, left maxillary sinus, and nasal and maxillary sinus airway complex after suture palatine expansion using the Hyrax disyuntor appliance with the digital measurement technique was carried out using Student’s paired *t*-test or the Wilcoxon rank test, and the comparison between operators was performed using the *t*-test or Mann–Whitney-Wilcoxon test for statistical analysis. The repeatability and reproducibility of these digital measurements were analyzed using Gage R&R statistical analysis. This analysis was conducted using the statistical analysis software SAS v9.4 (SAS Institute Inc., Cary, NC, USA) and R (R Foundation for Statistical Computing, Vienna, Austria). The statistical significance was *p* < 0.05.

## Results

Five patients (2 male) aged 10 to 13 years were consecutively selected and enrolled in the study. Table 1 shows the values for the mean and SDs for the preoperative and postoperative volumes, in addition to volume differences of the left maxillary sinus (mm^3^), after suture palatine expansion using the Hyrax disyuntor appliance.

**Table 1:** Descriptive statistics for the preoperative and postoperative volumes of the left maxillary sinus (mm^3^) and the volumetric differences observed in the left maxillary sinus (mm^3^) following suture palatine expansion using the Hyrax disyuntor appliance

	*n*	Mean	SD	Minimum	Maximum	*p*-Value
Preoperative left sinus	10	9023.30	5466.33	1775.00	17,175.00	
Postoperative left sinus	10	11,924.80	2831.06	8701.00	17,445.00	
Volumetric difference	10	2901.50	3325.10	137.00	8685.00	0.002

*SD* standard deviation

**Fig. 3:**
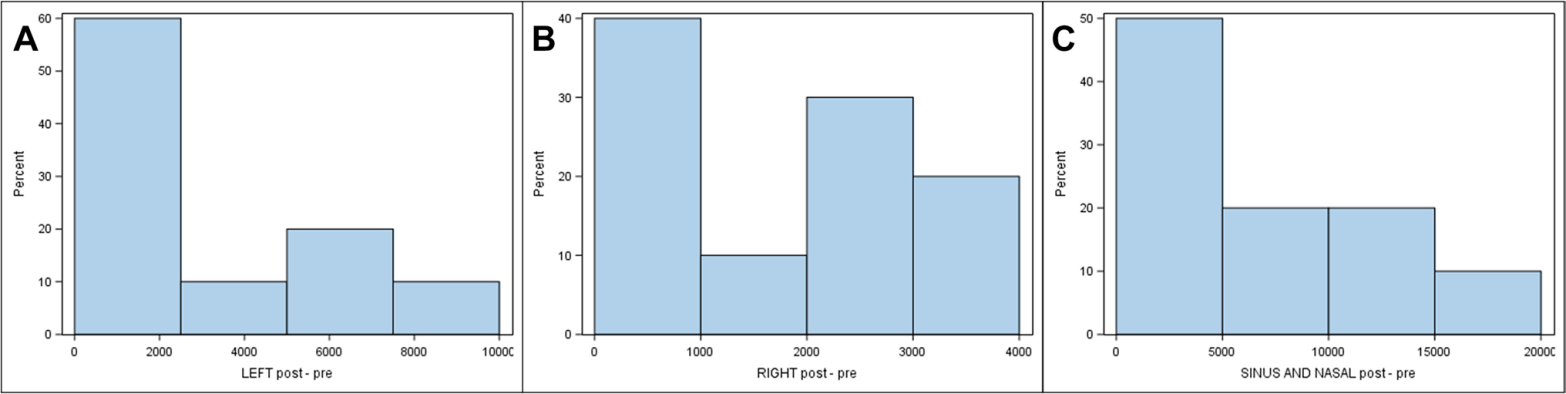
Histograms illustrate the distribution of differences in postoperative and preoperative values for the **A** left maxillary sinus, **B** right maxillary sinus, and **C** nasal and maxillary sinus airway complex. Positive values indicate higher values after suture palatine expansion

The paired *t*-test analysis showed statistically significant differences before and after intervention in the left maxillary sinus (mm^3^) after suture palatine expansion using the Hyrax disyuntor appliance (*p* = 0.002). The paired *t*-test did not reveal any statistically significant differences between operators with regard to the preoperative measurement volumes (*p* = 0.918), postoperative measurement volumes (*p* = 0.880), and the volumetric measurement differences of the left maxillary sinus (*p* = 0.765) after suture palatine expansion using the Hyrax disyuntor appliance (Fig. 3A).

Table 2 shows the means and SD values of the preoperative and postoperative volumes, as well as the difference in volume of the right maxillary sinus (mm^3^) after suture palatine expansion using the Hyrax disyuntor appliance.

**Table 2:** Descriptive statistics for the preoperative and postoperative volumes of the right maxillary sinus (mm^3^) and the volumetric differences observed in the right maxillary sinus (mm^3^) following suture palatine expansion using the Hyrax disyuntor appliance

	*n*	Mean	SD	Minimum	Maximum	*p***-**Value
Preoperative right sinus	10	13,613.60	3835.35	7697.00	19,712.00	
Postoperative right sinus	10	15,397.20	3535.62	10,160.00	21,073.00	
Volumetric difference	10	1783.60	1183.44	478.00	3556.00	0.001

*SD* standard deviation

Analysis with the paired *t*-test found statistically significant differences between the preoperative and postoperative volumes of the right maxillary sinus (mm^3^) after suture palatine expansion using the Hyrax disyuntor appliance (*p* = 0.001). The paired *t*-test did not reveal any statistically significant differences between operators with regard to the preoperative measurement volumes (*p* = 0.930), postoperative measurement volumes (*p* = 0.911), and the volumetric measurement differences of the right maxillary sinus (*p* = 0.532) after suture palatine expansion using the Hyrax disyuntor appliance (Fig. 3B).

Table 3 shows the means and SD values of the preoperative and postoperative volumes, as well as the difference in volume of the nasal and maxillary sinus airway complex (mm^3^) after suture palatine expansion using the Hyrax disyuntor appliance.

**Table 3:** Descriptive statistics for the preoperative and postoperative volumes of the nasal and maxillary sinus airway complex (mm^3^) and the volumetric differences observed in the nasal and maxillary sinus airway complex (mm^3^) following suture palatine expansion using the Hyrax disyuntor appliance

	*n*	Mean	SD	Minimum	Maximum	*p***-**Value
Preoperative nasal and maxillary sinus airway complex	10	36,098.30	7752.59	23,564.00	46,449.00	
Postoperative nasal and maxillary sinus airway complex	10	42,665.20	4185.42	37,963.00	51,316.00	
Volumetric difference	10	6566.90	5604.05	1570.00	17,628.00	0.005

*SD* standard deviation

Analysis using the paired *t*-test analysis found statistically significant differences between the preoperative and postoperative volumes of the nasal and maxillary sinus airway complex (mm^3^) after suture palatine expansion using the Hyrax disyuntor appliance (*p* = 0.005). The paired *t*-test did not show statistically significant differences between operators with regard to the preoperative measurement volumes (*p* = 0.831), postoperative measurement volumes (*p* = 0.951), and the volumetric measurement differences of the nasal and maxillary sinus airway complex (*p* = 0.804) after suture palatine expansion using the Hyrax disyuntor appliance (Fig. 3C).

**Fig. 4:**
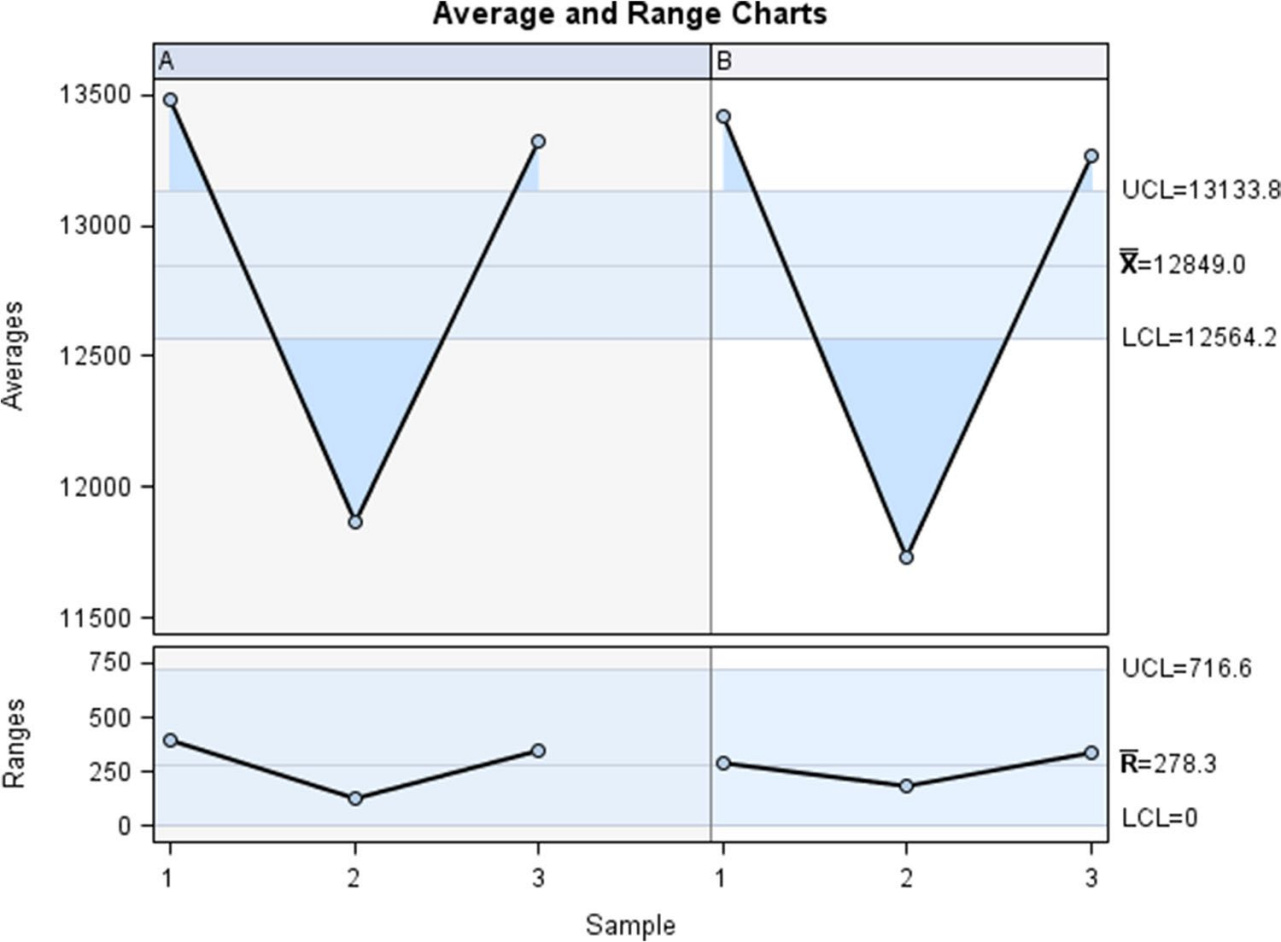
Average measures of the volume of the right maxillary sinus, left maxillary sinus, and nasal and maxillary sinus airway complex as measured by two operators

**Fig. 5:**
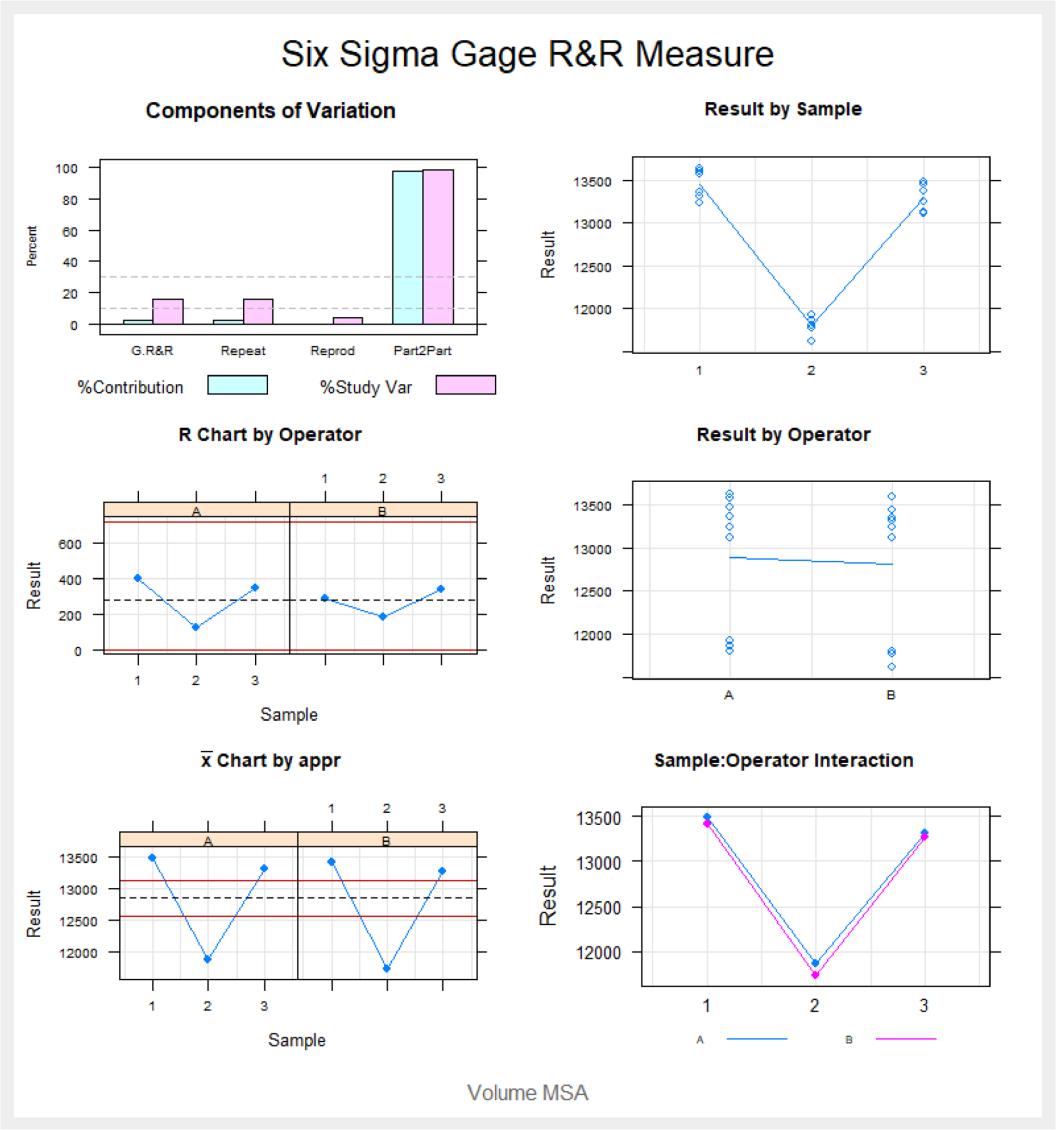
Analysis of measurement system for quantifying the volume of the right maxillary sinus, left maxillary sinus, and nasal and maxillary sinus airway complex with a chart showing the effect of each component on total variance (Components of Variation), a mean control chart, and a range control chart (R Chart by Operator and × Chart by appr), every measurement point in the graph (Trial by I and Trial by Operator), and the comparison between operators (**i**): Operator interaction. The abscissa axis expresses the volumetric values (mm.^3^)

Gage R&R statistical analysis of the digital measurement technique for analyzing the volume of nasal and maxillary sinus airways after suture palatine expansion using the Hyrax disyuntor appliance found that the variability attributable to the novel digital measurement method was 2.4% (among the measures of each operator) and 0.1% (among the measures of both operators), respectively, of the total variability across all samples. The digital measurement technique used to analyze the volume of the nasal and maxillary sinus airways after suture palatine expansion using the Hyrax disyuntor appliance was considered repeatable and reproducible because the variability scores were lower than 10%, which is considered repeatable and reproducible (Figs. 4 and 5).

## Discussion

The results of this study reject the null hypothesis (*H*_0_) stating that there is no difference in the volumes of nasal and sinus airways after suture palatal expansion using the Hyrax disyuntor appliance.

In the present study, the Hyrax disyuntor appliance was used to expand the palatine suture in young patients; however, this procedure also leads to an increase of the nasal cavity and maxillary sinus airways. Moxness et al. also reported that changes in the nasal cavity may contribute to development of obstructive sleep apnea [12]; however, Kim et al. showed that a decrease in maxillary sinus volume can also be considered a predictive factor for obstructive sleep apnea. Therefore, the authors of this study analyzed the volume of both the nasal cavity and the maxillary sinus [13]. Haas et al. described an increase in nasal cavity width between 2 and 4.5 mm, improving the perception of nasal breathing after radiological methods (RME procedures) [6]. Wertz et al. showed that RME procedures might improve the nasal airflow of patients affected by stenosis of the nasal airway; however, suture palatine expansion using RME procedures was not recommended for improving airflow [14].

Some studies have described spontaneous correction of nasal septum deviation following RME procedures [15]. Brown et al. reported that the deviated septum dropped down into the space created by laterally moving the maxilla process [16]. Previous studies have highlighted the impact of RME procedures on the nasal resistance airflow [7, 17]. Warren et al. assessed the nasal airway after suture palatine expansion and reported a nasal cross-section increase of between 45 and 55% after RME and SARME procedures [7], which remains stable for at least 1 year after suture palatine expansion using RME procedures [18]. The results of the present study found a nasal and maxillary sinus cross-section increase of 18.2%. These differences in results may be due to the measurement techniques used, as Warren DW analyzed the cross-sectional area of the nasal airway using a mathematical equation.

Additionally, several measurement methods have been proposed for assessing the dimensions of nasal cavity, including lateral and antero-posterior radiographs [6, 8]. However, these measurement methods are not useful for quantifying the airway volume of the nasal cavity after suture palatine expansion using RME procedures [19–22]. Montgomery et al. also used radiological methods, using computer tomography (CT) scans to analyze the nasal airway of human cadaver specimens, reporting that the CT scans provided a suitable measurement technique for assessing the nasal airway volume and a detailed imaging technique for assessing hard and soft tissue anatomy [10]. An interesting method with several studies to back it up involves using functional dynamic methods by way of acoustic rhinometry. The former method can be used to measure the dimensions of the nasal cavity by using acoustic waves to analyze the sound waves that reflect in the nasal cavity. Hahn et al. observed an increase of 10.13% in airway volume after suture palatine expansion using RME procedures, measured with the acoustic rhinometry method [9]. These results are different from those of the present study in the airway volume measurement technique and the disyuntor appliance (modified Haas disyuntor appliance) used. Babacan et al. reported a 13.8% increase in nasal volume after suture palatine expansion using RME procedures without a decongestant, measured using the acoustic rhinometry technique; however, the nasal airway volume increased by up to 15.16% with a decongestant [23]. Oliveira et al. used the acoustic rhinometry measurement method and reported an increase in nasal airway volume of up to 17.5% after suture palatine expansion using RME procedures using Haas, Hyrax, and bonded disyuntor appliances, with activation rates ranging from two turns per day to one turn per day and one turn every other day; the results obtained are very close to ones found in this study, even though the patients of this study were all subjected to RME procedures using the Hyrax disyuntor appliance [9]. Doruk et al. compared the results of the acoustic rhinometry measurement method with those from CT scans and reported high similarity between both measurement methods; furthermore, they stated that the decrease in the nasal resistance might be a result of intercanine width expansion [24]. Haralampidis et al. evaluated the effect of RME procedures on nasal cavity volume using CT scans and reported that nasal volume significantly increased (11.3%) [25].

Therefore, several digital measurement methods have been previously described for volumetric analysis of anatomical structures. Lotfi et al. studied changes in airway volume after different RME [26]. Their results differ from those obtained in the present study due to the expansion disyuntor appliance (bonded acrylic cap splint appliance), as well as the measurement method used. Finally, Timms et al. were able to demonstrate that the lateral walls of the nasal cavity move outward, expanding the width and volume of the cavity with a minimal cross-sectional area [27]. The triangular expansion pattern of the midpalatal suture results in greater width of the floor of the nasal cavity [28]. However, the patient’s perception of improved ability to breathe through the nose is not closely related to decreased resistance to nasal breathing [14].

The digital measurement is a novel technique that provides a simple, objective, and non-invasive measurement procedure for analyzing the volume of nasal and maxillary sinus airways after suture palatine expansion using the Hyrax disyuntor. However, a preoperative and postoperative CBCT scan and specific cephalometric software are necessary to perform the measurement analysis. This digital measurement procedure could be applied to further studies that require volumetric analysis, such in cases of sleep disordered breathing, where a CBCT may be indicated in order to check the improvement of the volume airway associated to the growth pattern, skeletal class, or age of the patient. This could present a new treatment approach as an alternative to surgery in moderate cases of hypopnea in children.

The nasal cavity is a very complex structure. It is extremely difficult to measure separately due to the difficulty to isolating it in the drawing, as it overlaps the maxillary sinus in many cases, and the drawing is a two-dimensional attempt to portray a three-dimensional structure. As a result, the whole nasal and sinus complex was included in the measurement of the nasal cavity in order to simplify the measurement process. If our concern is solely the nasal cavity, it is advisable to measure the maxillary sinuses separately, which is a relatively simple procedure; then measure the whole nasal-sinus complex; and find the difference between the two. Additionally, until now, there are not any studies that have not found differences in volume or width of the nasal cavities or maxillary sinus after rapid maxillary expansion.

## Conclusion

In conclusion, within the limitations of this study, the results indicate that the proposed digital technique is repeatable and reproducible for analyzing the volume of the nasal and maxillary sinus airways after suture palatine expansion using the Hyrax disyuntor.

## Data Availability

The datasets collected and analyzed during the present study can be made available upon reasonable request by contacting the corresponding author.
